# Older African Americans with the ABCA7‐80 high‐risk genotype have diminished slow oscillation power during non‐REM sleep

**DOI:** 10.1002/alz.091404

**Published:** 2025-01-09

**Authors:** Payton G. White, Miray Budak, Alyssa Arnasalam, Mustafa Sheikh, Bernadette A. Fausto, Mark A. Gluck

**Affiliations:** ^1^ Rutgers University–Newark, Newark, NJ USA

## Abstract

**Background:**

African Americans are among the most vulnerable demographic groups to both sleep deficiencies and Alzheimer’s disease (AD)3. ABCA7‐80 (rs115550680) known as adenosine triphosphate (ATP)‐binding cassette member 7, plays a role in the transport of amyloid precursor protein, clearance of cellular Aβ, and lipid metabolism: three processes associated with late‐onset AD2. Slow oscillations, which characterize non‐REM sleep, are implicated in waste clearance and memory consolidation in the brain1. The present study investigated the putative association between ABCA7‐80 risk on non‐REM slow wave oscillations among cognitively unimpaired older African Americans.

**Method:**

Participants were drawn from the ongoing longitudinal study, Pathways to Healthy Aging in African Americans conducted at Rutgers University–Newark. 75 participants, ages 60‐87 years old completed a saliva test for genotyping and underwent at‐home sleep monitoring over two nights using the DREEM 3 Headband. MANCOVA statistical analysis was performed on the sample data.

**Result:**

Individuals with the ABCA7‐80 high‐risk allele, had significantly lower frontal slow oscillation relative power than individuals with the non‐risk allele (F= 2.175, η 2= 0.137, p= 0.084).

**Conclusion:**

This preliminary data shows that individuals who have the ABCA7‐80 high‐risk genotype may have lower slow oscillation relative power. This holds importance for AD in African Americans as there is evidence that ABCA7‐80 is more relevant for cognitively unimpaired older African Americans.

